# Application of the Athlete's Performance Passport for Doping Control: A Case Report

**DOI:** 10.3389/fphys.2018.00280

**Published:** 2018-03-29

**Authors:** Sergei Iljukov, Stephane Bermon, Yorck O. Schumacher

**Affiliations:** ^1^KIHU - Research Institute for Olympic Sports, Jyvaskyla, Finland; ^2^LAMHESS, University Cote d'Azur, Nice, France; ^3^IAAF Health and Science Department, Monaco, Monaco; ^4^Aspetar Orthopaedic and Sports Medicine Hospital, Doha, Qatar

**Keywords:** blood doping, doping in sports, critical speed, performance, target testing

## Abstract

The efficient use of testing resources is a key issue in the fight against doping. The longitudinal tracking of sporting performances to identify unusual improvements possibly caused by doping, so-called “athlete's performance passport” (APP) is a new concept to improve targeted anti-doping testing. In fact, unusual performances by an athlete would trigger a more thorough testing program. In the present case report, performance data is modeled using the critical power concept for a group of athletes based on their past performances. By these means, an athlete with unusual deviations from his predicted performances was identified. Subsequent target testing using blood testing and the athlete biological passport resulted in an anti-doping rule violation procedure and suspension of the athlete. This case demonstrates the feasibility of the APP approach where athlete's performance is monitored and might serve as an example for the practical implementation of the method.

## Introduction

The true prevalence of doping use in elite sports is unknown, but studies give estimates between 14 and 39% (de Hon et al., 2015). According to the World Anti-Doping Agency (WADA)'s statistics, approximately 2% of the collected doping samples yearly are reported to contain a banned substance. The figure remains quite stable despite the gradual increase of both, the number of doping tests conducted and the sensitivity of the analytical methods (Marclay et al., 2013). Based on these findings, a substantial discrepancy between the estimated prevalence of doping and the number of positive doping cases remains.

The WADA is aware of the issue and demonstrates efforts toward its solution. One of the major changes in recent years was the paradigm shift from purely chemical analyses and detection of banned substances in biological samples to serial analysis of indirect biomarkers demonstrate the usage of forbidden substances or methods. This culminated in concept of Athlete's Biological Passport (ABP) that longitudinally tracks certain biomarkers for features of doping (Sottas et al., 2011). In addition to the sanctioning of more than 100 athletes based on their individual profiles, the introduction of the ABP also resulted in an increased number of positive doping cases for erythropoiesis stimulating agents (ESA) using traditional anti-doping methods, due to better test targeting based on information from the ABP (Zorzoli and Rossi, 2012).

The original idea of “athlete's performance profiling” or monitoring individual performances for better informed decisions on doping testing has been presented by Schumacher and Pottgiesser (2009). The main objective of performance profiling or “athlete performance passport” (APP) in sport is to distinguish between consistent and unexpectedly disproportionate performances. Excellent performance itself is not a proof of any wrongdoing or doping. However, through longitudinal monitoring, inconsistently excellent performance could be a warning sign that needs further attention from anti-doping authorities.

The purpose of the present case report is to illustrate the feasibility of this approach. In this example, the longitudinal monitoring of performance identified a suspicious athlete who was subsequently successfully target tested and convicted of an anti-doping rule violation.

## Subjects and methods

A pilot project was initiated for middle- and long-distance runners by a National Anti-Doping Organization (NADO). The athletes were included in the NADOs testing pool and its ABP testing program. The rationale for inclusion in the registered testing pool were based on athlete's age, gender, ethnical background, years of training, club, coach, and performance level.

Individual APP were created for the athletes. The APP consisted of all accessible competition results in long- and middle-distance events. Results were gathered from an open database www.tilastopaja.com. Based on the available competition data, critical speed (CS) calculations (Vanhatalo et al., 2011) were performed as follows in order to determine the physiological capacities of each athlete.

The relationship between running speed and exercise duration is the core of so-called critical power concept. The “critical power” determines a performance (for instance running speed) an athlete is able to sustain for a given amount of time. It is expressed as the equation:

$$T=\left(D-W^{\prime }\right)/CS$$

Where, *T* is time (s), *D* is the covered distance (m), *W*′ is anaerobic work capacity (m), and *CS* is the *CS* (m/s). When *D* and *T* are known for two distinct competitions, then *W*′ and *CS* can be calculated. The previous competitions results were used to predict performances at future events (running speed/competition times over defined distances). After each event, the results obtained in reality were compared to the estimated performance and matched to the blood profile.

A number of factors, e.g., race tactics, pacing, and environmental conditions, may affect final competition results. Hence, Hopkins and Hewson have reported a possible intra-individual variability of 1.0–1.4% in elite endurance runners (Hopkins and Hewson, 2001). Timing for blood testing was then individually adapted based on the competition schedule and the results of the athletes. The written consent and permission for this publication from the athlete was acquired.

## Case report

On day 1, the athlete reported in the present study ran a half-marathon in a time close to his previous personal best i.e., 1 h 08 min 15 s (see Table 1). This result was in line with his historical results over various distances in previous years.

**Table 1:** Athlete's performance results calendar and calculations.

**Day**	**Competition**	**Distance (m)**	**Time (s)**	**Average Speed (m/s)**	**Races used for calculation of CS**	**Predicted CS (m/s)**	**Predicted time (s)**	**Deviation of competition result from prediction (%)**
1	Half-marathon	21,097.5	4,095	5.15	D1–D60	4.82	4,095	0
60	Road race	12,000	2,209	5.43			2,209	0
78	Marathon	42,195	8,450	4.98			8,469	+0.22
147	Half-marathon	21,097.5	4,438	4.75	D60–D78	4.84	4,089	−8.53
161	Half-marathon	21,097.5	4,214	5.01			4,089	−3.05
204	Road race	10,000	1,899	5.27			1,796	−5.76
239	Marathon	42,195	8,251	5.10	D161–D204	4.79	8,615	+4.22

**Figure 1:**
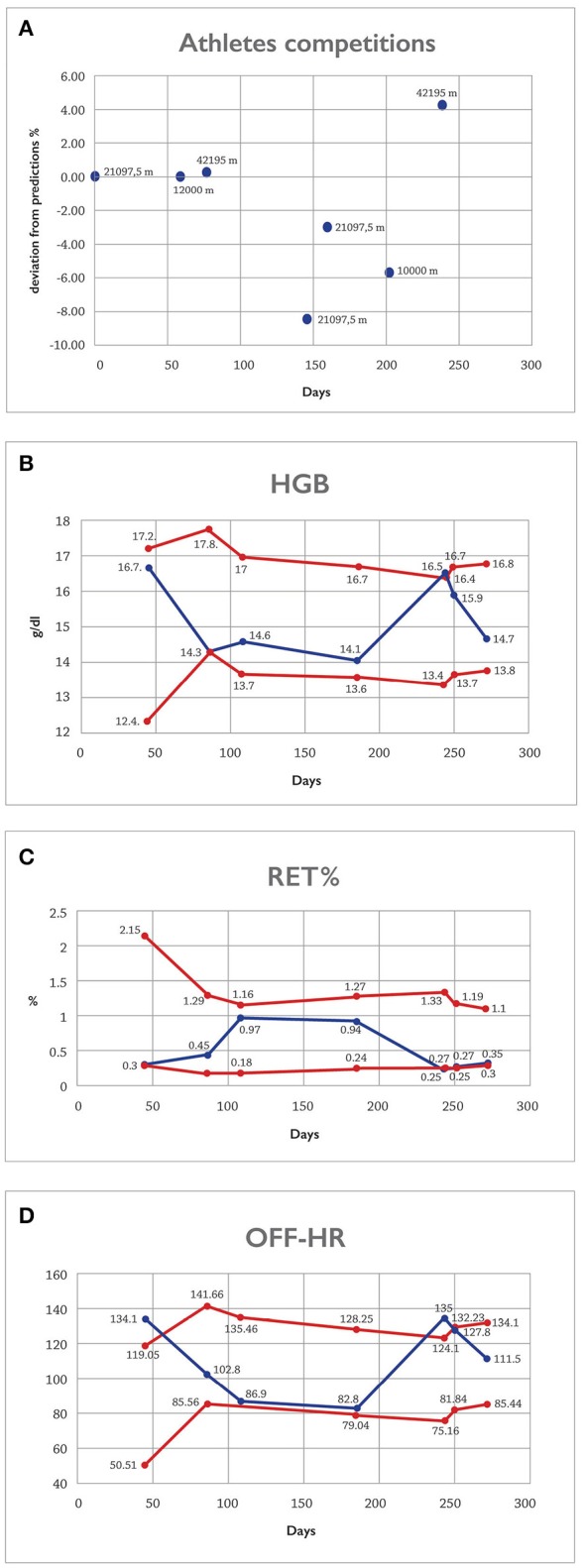
On the horizontal axis, the observation period is pictured in days. **(A–D)** The normalized deviation from the expected value based on CS calculations **(A)** and the blood data of the athlete based on the adaptive model of the ABP **(B–D)**. In panel **(A)**, a positive deviation indicates a better than predicted performance. The red lines in panels **(B–D)** illustrate the individually calculated reference limits of the ABP for each variable. The blue lines represent the data.

The first blood sample was taken on day 45 of the observational period as a routine out of competition test and, for logistical reasons, the second test was only obtained on day 86 (Figure 1). The discrepancy in hemoglobin, reticulocyte parameters, and high OFF score between the first and second samples raised attention and vigilance to follow his performances closer. Analysis of competition results for the period between the two blood sampling showed that at day 60 of the follow-up athlete ran his personal best at the 12 km race (Table 1). On day 78 the athlete achieved his best personal marathon time (2 h 20 min 50 s) which was in line with the calculated predicted time i.e., 2 h 21 min 8 s. This calculation was based on the previous half-marathon time (day 1), and 12 km road race time (day 60) (see Calculation 1 below). Further when choosing distances for the calculations we divided results seasonally into spring, summer and autumn trimesters and used the last two competitions of the trimester for predictions of coming trimester.

### Calculation 1

Prediction of performance time *T*_*i*_ of distance 42,195 m by using length (m) of distance *D*_1_ = 21,097.5 m and distance *D*_2_ = 12,000 m and performance time (s) of distances *D*_1_ and *D*_2_, that is *T*_1_ 4,095 and *T*_2_ 2,209 s accordingly.

$$CS=\frac{21,097.5\;-\;12,000}{4,095\;-\;2,209}=4.824\text{ m/s}$$

$$W^{\prime }=\frac{12,000\;\times \;4,095\;-\;21,097.5\;\times \;2,209}{4,095-2,209}=1,344.44\text{ m}$$

$$T_{42,195}=\frac{42,195\;-\;1,344.44}{4.824}=8,468.2\;s=2\text{ h }21\text{ min }8\text{ s}$$

Following this event, the next three competition performances between days 147 and 204 were also consistent with (or worse than) predictions and without major discrepancies compared to previous year's results in the same competitions and no need for further targeted testing was seen (Table 1, Figure 1). At the end of the season however, on day 239, the athlete participated in an international level marathon, where he finished again with a new personal best time of 2 h 17 min 31 s. Importantly, that with this competition result, the athlete qualified for major international competition and was selected for the national team. Based on the athletes' results of the latest half-marathon on day 161 and the 10 km road race result on day 204, and using the critical power concept, the athlete's predicted time for the marathon was calculated to be 2 h 23 min 35 s (Calculation 2).

### Calculation 2

$$CS\;=\;\frac{21,097.5-10,000}{4,214\;-\;1,899}=\;4.794\text{ m/s}$$

$$W^{\prime }\;=\frac{10,000\;\times \;4,214\;-\;21,097.5\times 1,899}{4,214\;-\;1,899}=896.694\text{ m}$$

$$T_{42,195}=\frac{42,195\;-\;896.694}{4.794}=8,614.6\;s=2\text{ h }23\text{ min }35\text{ s}$$

These races were chosen as less intra-individual variability for road races and changes in physical performance within a given timeframe are expected. Thus, the actual competition time was markedly beyond predicted time with a difference of 6 min 4 s. This represented a deviation from prediction of 4.22% and was exceedingly better than would be expected. At this point the decision to collect additional blood samples for the purpose of the hematological profile of the ABP was taken (Figure 1). Subsequent targeted samples for biological passport (days 243 and 250) demonstrated clear features of blood doping. Following IAAF disciplinary procedures, the athlete admitted the anti-doping rule violation and was banned from competition.

## Discussion

Optimally targeted anti-doping tests are crucial for the success and the cost-efficiency ratio of any anti-doping program. Considering the high number of athletes to be controlled by National Anti-Doping Agencies it is crucial to prioritize and direct anti-doping efforts primary to high risk groups and individuals. Thus, decision on testing must involve multifactorial risk assessment that includes a broad spectrum of information (Schumacher and Pottgiesser, 2009; Saugy et al., 2011; Marclay et al., 2013).

Considering that the main goal of doping is to improve athlete's performance, it seems reasonable to systematically monitor athletes' competition results for identification of possible irregularities and initiation of additional anti-doping actions if required. As the biological passport experience demonstrated, in addition to sanctioning, longitudinal monitoring of athlete's blood parameters also helps to reveal suspicious traits that could lead to targeted testing and cases of anti-doping rule violation (Zorzoli and Rossi, 2012). Especially in the present case, where the manipulation was likely the application of a blood transfusion (visible in samples 5 and 6), correct timing of the tests is the key. In fact, if the samples had not been obtained within a certain timeframe, the typical features would not have been visible anymore. With the anticipated competition schedule and the previous results of the athlete at hand, it was much easier to time the tests accordingly.

There is a lack of longitudinal studies on performance changes over competitive season on highly trained runners. In recent study Galbraith et al. (2014) found CS to be sensitive and reliable measure of performance changes over competitive season. There was statistically significant change in CS over competitive season and tendency for change when evaluated on 84 day periods. When choosing races for calculations we divided results seasonally into spring, summer, and autumn trimesters and used the last two competitions of trimester for predictions of the coming trimester. This division is somehow close to 84 days periods of Galbraith et al. study, thus allowing adjust calculations even for slight changes in CS during competitive season.

It must be stressed that any discrepancy between predicted and actual competition performance should not be seen as an indication of doping *per se*, as there are a number of factors that obviously affect performance predictions and measurements. Intra-individual variability in performance results might be influenced by pacing, tactics, route of the race and environmental conditions. Other factors that might impact performance, such as tapering, are not taken into account in the current modeling, as it assumed that these factors will be similar in all races. The possibility of flawed competition data by previous doping must also be considered. However, some important variation or deviations of performance prediction are to be considered as a “red flag” warning that needs further evaluation and closer scrutiny from anti-doping authorities.

This case nevertheless demonstrates the rationale for an APP and an example of its potential application for targeted doping testing. The APP could be potentially used as an additional tool together with the longitudinal monitoring of biological parameters for improved targeting of suspicious athletes and increasing the quality of anti-doping testing activities and thus use available resources more efficiently. Although certainly not the only existing mathematical model, the CS concept seems to be a promising one to be used in the APP. There is urgent need for further research on performance passport-related topic. A better knowledge of inter- and intra-individual performance variability or performance evolution during the whole athletic career is also warranted for different sports and events in order to establish meaningful alarm thresholds for the APP.

## Author contributions

Conception of the publication and preparation of the manuscript: SI, YS, and SB. All authors read and approved the final manuscript.

### Conflict of interest statement

The authors declare that the research was conducted in the absence of any commercial or financial relationships that could be construed as a potential conflict of interest.
